# The complete mitochondrial genome of the freshwater fairy shrimp *Branchinella kugenumaensis* Ishikawa 1894 (Crustacea: Anostraca: Thamnocephalidae)

**DOI:** 10.1080/23802359.2020.1721367

**Published:** 2020-02-03

**Authors:** Ri-Sheng Yang, Yi-Tao Chen

**Affiliations:** College of Life Sciences, Zhejiang Chinese Medical University, Hangzhou, Zhejiang, China

**Keywords:** Mitogenome, *Branchinella kugenumaensis*, Anostraca, Thamnocephalidae

## Abstract

In this study, we determined and analyzed the complete mitochondrial genome of the freshwater fairy shrimp *Branchinella kugenumaensis* Ishikawa 1894 (Crustacea: Anostraca: Thamnocephalidae). The mitogenome is 15,127 bp in length, consisted of 37 genes that participate in protein production and energy metabolism of mitochondria. The gene order of the *B. kugenumaensis* mtDNA exhibits major rearrangements compared with the pancrustacean ancestral pattern or other known anostracan mitogenomes, representing a novel mitochondrial genomic organization within the Crustacea. A maximum-likelihood phylogenetic analysis based on concatenated nucleotide sequences of protein-coding genes places *B. kugenumaensis* next to *Streptocephalus sirindhornae*, inside the Anostraca clade. Our study will provide new evidence to the less sampled anostracan evolution and take a further step to the completion of the Branchiopoda tree of life.

The freshwater fairy shrimp *Branchinella kugenumaensis* is distributed in ephemeral waters in southeast Asia (Belk and Brtek 1995), such as temporary pools by snowmelt and artificial irrigation in rice fields. Like most branchiopod crustaceans, *B. kugenumaensis* undergoes a typical seasonal lifestyle of fast rates of growth/maturation/reproduction in favorable seasons while producing dormant resting eggs in unfavorable seasons (Huang et al. 2010).

Adult individuals of *B. kugenumaensis* were collected from a rice field (119°38′ E, 29°05′ N) in Jinhua, Zhejiang Province, China. The voucher specimen was deposited at Zhejiang Chinese Medical University (No. BKU-ZJ-001). Total genomic DNAs of *B. kugenumaensis* were extracted from 15 adults using the proteinase K/phenol/chloroform method. We amplified and sequenced the partial *cox1* and *rrnL* fragments primed by LCO1490/HCO2198 and 16Sar/16Sbr, respectively. Based on the sequences acquired, we designed nested gene-specific primers and amplified longer mtDNA portions by high-fidelity PCR. The two fragments (about 5 and 10 kb in length) were purified, sonicated, constructed into shot-gun DNA libraries and randomly sequenced. The complete mitochondrial genome was assembled, annotated, and then submitted to GenBank under the accession number MN660045.

The mitochondrial genome of *Branchinella kugenumaensis* is a 15,127 bp circular dsDNA molecule, comprising 13 protein-coding genes, 2 ribosomal RNA genes, and 22 transfer RNA genes plus one putative control region, representing a typical composition for metazoan mitogenomes. Two major adjacent overlaps appear between *atp8* and *atp6*, as well as between *nad4* and *nad4L*, where seven and eight nucleotides are shared, respectively. The protein-coding genes have five types of start codons (4 ATGs, 4 ATTs, 3 TTGs, 1 ATA, and 1 GTG) and three complete or incomplete stop codons (4 TAAs, 2 TAs, and 7 Ts).

To date, gene arrangements of all determined branchiopod mitogenomes exhibit two patterns: a ‘pancrustacean ancestral pattern’ (Cook et al. 2005) shared by four Phyllopoa species (*Daphnia magna*, *D. pulex*, *Triops cancriformis,* and *T. longicaudatus*) and the other shared by six Anostraca species (four *Artemia* species, *Phallocryptus tserensodnomi,* and *Streptocephalus sirindhornae*). Based on the pancrustacean pattern, the anostracan pattern translocates (*trnI*+*trnQ*) from between the control region and *trnM* to between *trnW* and *trnC*, together with an inversion of *trnI*. The *B. kugenumaensis* mitogenome underwent a novel major change from the latter pattern, where the portion (*trnM*+*nad2*+*trnW*+*trnI*) entirely inversed from one strand to the other.

Nucleotide sequences of all 13 protein-coding genes from *B. kugenumaensis* and other 10 branchiopod mitogenomes were concatenated, multi-aligned, and subjected to a maximum-likelihood phylogenetic analysis. The inferred topology places *B. kugenumaensis* inside the Anostraca clade (Figure 1), neighboring another freshwater fairy shrimp *Streptocephalus sirindhornae* (Liu et al. 2016). The phylogenetic relationship is consistent with the widely-known species tree (Martin and Davis 2001).

**Figure 1.**
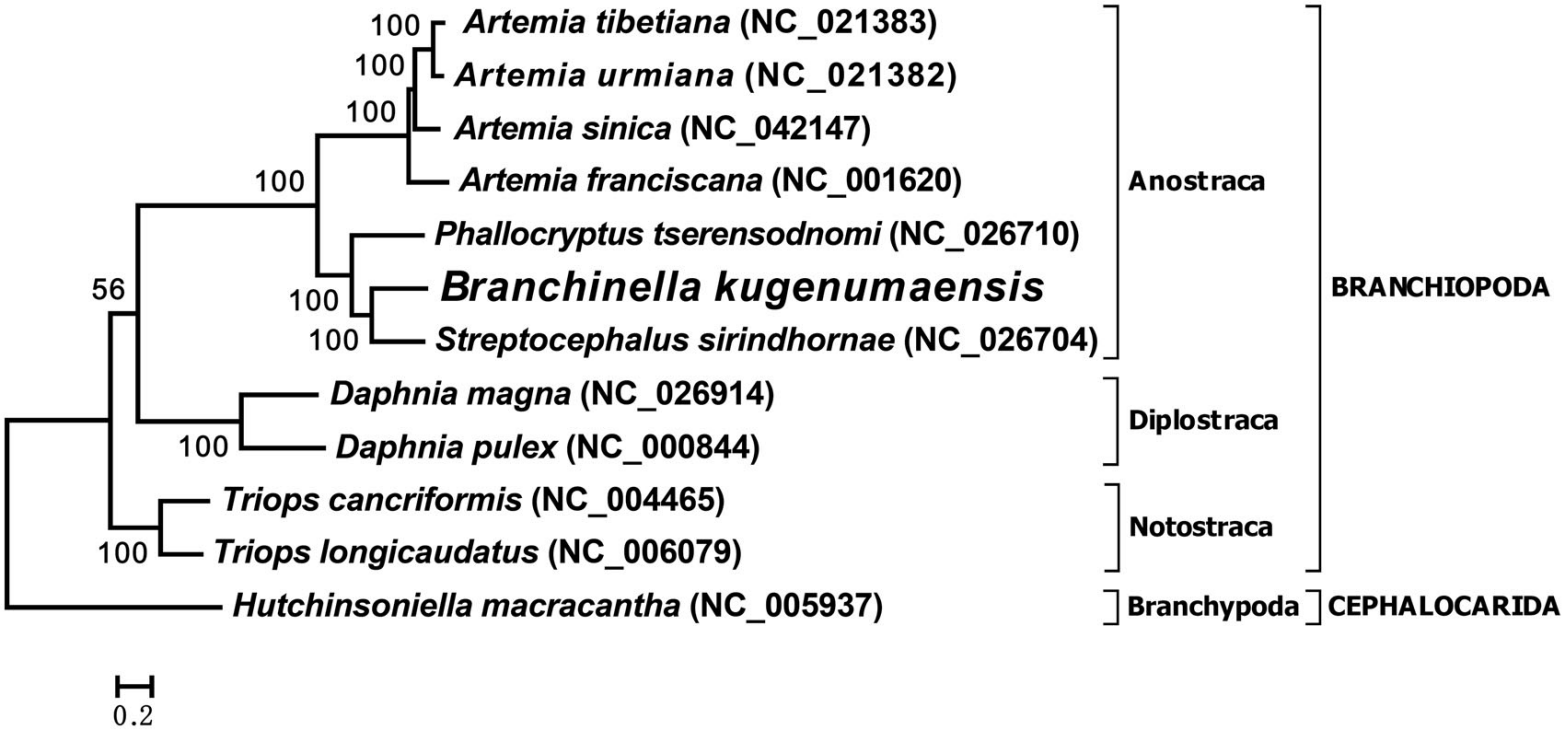
Phylogenetic analysis of the *Branchinella kugenumaensis* mitogenome. Concatenated nucleotide sequences of 13 protein-coding genes were multi-aligned with ClustalW and subjected to maximum-likelihood phylogenetic analysis implemented in MEGA7. GTR + G+I was selected as the evolutionary model and 1000 replications of bootstrapping was performed to test the phylogeny (supporting percentages besides nodes). The horseshoe shrimp *Hutchinsoniella macracantha* served as an outgroup. GenBank accession numbers of each mitogenome are marked in parentheses. The tree is drawn to scale, with branch lengths measured in the number of substitutions per site.

Anostracans of the Branchiopoda occupy primitive taxonomic levels in the evolution of crustaceans, an animal group with vast richness of diversity and invaluable importance of economy. However, the extremely imbalanced sampling constraints our understanding of their evolutionary history. We hope that our study can make contribution to the completion of this important part of The Tree of Life.
